# Renal Infarction Incidence, Risk Factors, and Risk of Mortality and KRT: A Retrospective Cohort Study

**DOI:** 10.34067/KID.0000000742

**Published:** 2025-02-18

**Authors:** Hicham I. Cheikh Hassan, Karumathil M. Murali, Kelly Lambert, Sola Aoun Bahous, Siba Kalab, Hala Kilani, Judy Mullan

**Affiliations:** 1 School of Medicine, Lebanese American University, Beirut, Lebanon; 2 Faculty of Science, Medicine and Health, Graduate School of Medicine, University of Wollongong, Wollongong, New South Wales, Australia; 3 Department of Renal Medicine, Wollongong Hospital, Wollongong, New South Wales, Australia; 4 Faculty of Science, Medicine and Health, School of Medicine, Indigenous and Health Sciences, University of Wollongong, Wollongong, New South Wales, Australia

**Keywords:** dialysis, kidney failure, mortality risk, renal replacement therapy

## Abstract

**Key Points:**

- This study examined the incidence, risk factors and characteristics of renal infarction in a retrospective 10-year follow-up population cohort.
- Renal infarction was not associated with an increased risk of mortality compared with the general population when adjusted for age and comorbidities.
- However, there was an increased risk of kidney failure requiring KRT.

**Background:**

Renal infarction is a rare medical condition, with most studies focusing on prevalence and risk factors. Very few studies examined long-term outcomes and, to the best of our knowledge, there are no studies comparing patients with renal infarction to a control group for long-term outcomes such as mortality or KRT. We used a hospital population database to examine incidence, prevalence, and risk factors for renal infarction and long-term outcomes such as mortality and KRT, comparing patients with renal infarction to the general population and to a matched cohort.

**Methods:**

The retrospective cohort study of adult patients from an Australian Local Health (2008–2017). We examined the incidence and prevalence of renal infarction along with risk factors associated with a renal infarction. We compared patients with a renal infarction to the general population and a 1:5 matched cohort to examine risk of mortality and KRT. Logistic regression was used to examine risk factors for renal infraction with odds ratio (OR) and 95% confidence intervals (CIs), and Cox proportional hazard regression was used to determine hazard ratio (HR).

**Results:**

Of the 140,099 patients included, a renal infarction occurred in 119 (0.1%) with a crude incidence of 1.9 per 10,000 patient-years (95% CI, 1.6 to 2.2). Factors associated with the risk of a renal infarction were age (OR, 1.17 by decade; 95% CI, 1.06 to 1.30), atrial fibrillation (OR, 2.55; 95% CI, 1.54 to 4.28), valvular heart disease (OR, 2.88; 95% CI, 1.26 to 6.57), and peripheral vascular disease (OR, 7.69; 95% CI, 4.54 to 13.4). During follow-up, there was no increased risk of mortality after adjusting for age and comorbidities (HR, 1.19; 95% CI, 0.89 to 1.61). However, the risk of KRT was significantly elevated even after adjusted for confounder (HR, 8.4; 95% CI, 3.6 to 19.7). These results remained valid in the matched analysis.

**Conclusions:**

Renal infarction is an uncommon event. The risk of mortality is not significantly increased when compared with the general population after adjusting for confounders. However, the risk of KRT remains significantly increased.

## Visual Abstract

**Figure:**
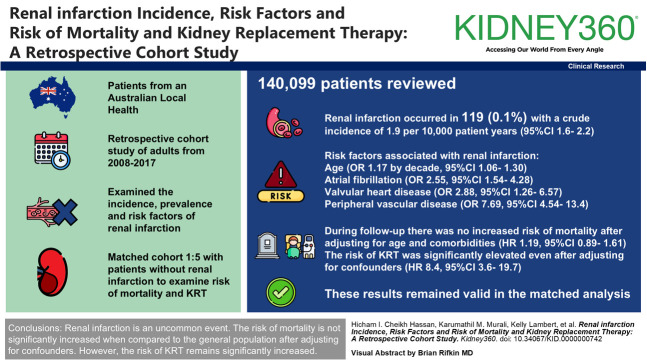

## Introduction

Renal infarction is a rare condition defined by ischemic damage of the renal parenchyma caused by the sudden interruption of blood flow in the renal arteries or their branches. It is a rare medical condition, with an incidence rate estimated to range from 0.004% to 1.8% depending on the population studied.^1–5^ The most common clinical presentation of renal infarction is acute abdominal or flank pain.^1–4,6–8^ However, symptoms can be nonspecific and may only include nausea, vomiting, fever, and even atypical presentations, such as confusion, diarrhea, or dyspnea.^3,4,7,8^

Causes of renal infarction are diverse and have traditionally been divided into renal artery origin, cardiac origin, hypercoagulable disorders, or idiopathic (or uncertain) origin.^9,10^ Renal artery causes include conditions such as atherosclerosis, aneurysm, dissection, fibromuscular dysplasia, vasculitis, and trauma. Although cardiac causes primarily involve thromboembolism resulting from atrial fibrillation or endocarditis,^9–11^ idiopathic causes, or causes of uncertain origin, account for 25%–30% of all presentations^9–12^ and bilateral kidney involvement can be seen in 20%–25% of presentations.^9,12–14^

The rarity of the condition, combined with a vague clinical presentation and diverse etiologies, often leads clinicians to initially consider more common differentials such as pyelonephritis, urolithiasis, or other abdominal disorders. This can result in either misdiagnosis or delays in diagnosis, potentially leading to inappropriate management or to delays in the initiation of appropriate treatment. Consequently, ongoing and/or irreversible kidney damage may take place.

Almost all studies examining renal infarction are retrospective, single-center cohort in design. These studies have primarily focused on the prevalence of renal infarction within specific populations, baseline comorbid conditions, clinical presentation, and underlying causes of renal infarction. However, very few studies have examined long-term outcomes. It is important to note that, to date, there is a paucity of studies examining the risk of mortality or the development of kidney failure in patients with kidney infarction, especially when it comes to comparing these outcomes with those in a normal or matched population.

To address the existing gap in the literature, we used an Australian Local Health District hospital population database. Our study aimed to investigate renal infarction to determine (*1*) the incidence and prevalence of renal infarction over a 10-year period, (*2*) the baseline comorbid risk factors associated with renal infarction, and (*3*) long-term outcomes of renal infarction, including mortality and kidney failure requiring dialysis. These outcomes were compared with those of the general population and a matched cohort.

## Methods

### Study Cohort

A retrospective population-based cohort study was performed using longitudinal data from an Australian regional health service, the Illawarra Shoalhaven Local Health District (ISLHD). We included adult patients (18 years or older) who presented to the ISLHD hospitals or emergency departments or who had laboratory tests using the ISLHD laboratory services between January 1, 2008, and December 31, 2017. This study was approved by the ISLHD/University of Wollongong Human Research Ethics Committee (2018/409) conformed to the Declaration of Helsinki and conducted in accordance with Strengthening the Reporting of Observational Studies in Epidemiology guidelines.^15^

We excluded patients who (*1*) were nonresidents of the ISLHD, (*2*) commenced KRT before the start of the study period, (*3*) were diagnosed with a renal infarct after commencing KRT, (*4*) were followed up for <3 months, (*5*) had incomplete patient information, and (*6*) were older than 95 years.

### Data Sources and Baseline Variables

Routinely collected deidentified existing data were extracted from the Illawarra Health Information Platform and used for this study. Details about the Illawarra Health Information Platform and the cohort database have been previously described.^16^ The KRT start date was determined by data-linkage with the Australian and New Zealand Dialysis and Transplantation registry.

Baseline variables were sex, age (at first presentation), urbanity (major city or regional), and socioeconomic indices for areas (measured by Index of Relative Socioeconomic Advantage and Disadvantage and categorized into tertiles of high, middle, and low, with higher scores reflecting higher socioeconomic status). Urbanity and Index of Relative Socioeconomic Advantage and Disadvantage were determined from the postcode of the patients' residential addresses, using the Accessibility/Remoteness Index of Australia taken from the Australian Bureau of Statistics using Australian Standard Geographical Classification from census data.^17^ Urbanity was defined as a major city if the population of the area was 100,000 individuals or more; otherwise, the urbanity was defined as regional. For each patient, a baseline e-phenotype was created using comorbidities based on the International Classification of Diseases-Tenth (ICD-10) Revision codes from hospitalized admissions (Supplemental Table 1). CKD was determined from serum creatinine on the basis of the Kidney Disease Improving Global Outcomes definition^18^ and subgrouped as per eGFR into <30, 30–60, and >60. AKI was also determined from a serum creatinine measurement compared with a baseline level as per Kidney Disease Improving Global Outcomes definitions and grouped according to severity into stage 1, 2, or 3.^19^ Both AKI and CKD definitions have been previously described in detail for our cohort in previous publications.^16,20^

### Definitions

Our exposure was an episode of renal infarction based on International Classification of Diseases, Tenth Revision coding (N28.0). Our primary outcome was mortality with a secondary outcome of kidney failure requiring KRT (hemodialysis, peritoneal dialysis, or transplantation). Follow-up was until mortality, KRT, last follow-up visit, or end of the study period.

Given the significant comorbid burden commonly observed in patients with renal infarction and the possibility that outcomes may reflect this high baseline disease burden, we also undertook a further analysis by matching patients with renal infarction to those with no renal infarction in a 1:5 ratio. Matching was based on age (per 5 years), sex, CKD, hypertension, diabetes, coronary artery disease, congestive heart failure, cerebrovascular disease, and atrial fibrillation.

### Statistical Analysis

For baseline characteristics, categorical data are expressed as numbers (percentages) and compared using the chi-squared test. Continuous data are expressed as a mean with SD or a median with an interquartile range and analyzed as per distribution using the *t* test and the Mann–Whitney *U* test. The crude incidence with 95% confidence intervals (CIs) of renal infarction per 10,000 population was examined for the total patient population and by sex and age group (younger than 50 years, 50–70, and older than 70 years). Baseline risk factors for an episode of renal infarction were examined using logistic regression. The multivariate model was refined through backward selection, starting with the inclusion of variables that demonstrated *P* values of <0.10 in the univariate analysis (age was retained in the analysis as it was deemed clinically meaningful and significant). The association between renal infarct and outcomes, such as mortality or KRT, compared with the nonrenal infarct population was examined using Cox proportional hazard regressions. Time to renal infarction from entry into the study was specified as a time-varying exposure. Patients with no renal infarction served as the reference group with the results expressed as a hazard ratio (HR) with 95% CIs. Analysis was performed for the whole population and for the 1–5 matched cohort population using several models as follows: (*1*) model 1: unadjusted, (*2*) model 2: included baseline characteristic (age, sex, urbanity, and socioeconomic status), and (*3*) model 3: model 2+comorbidities determined to influence outcome. All analyses were preformed using STATA (version 15.1).

## Results

### Study Population

**Figure 1:**
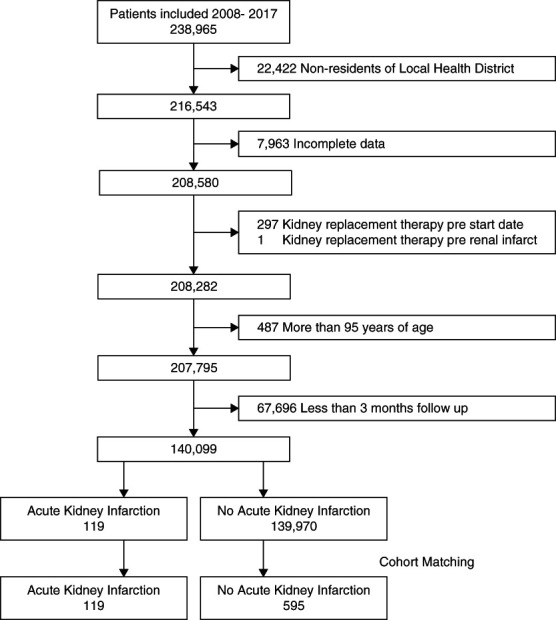
**Cohort selection flow diagram.** Serial exclusion criteria to derive the final study cohort and matched cohort.

A total of 238,965 patients met our inclusion criteria, with our final cohort after exclusions consisting of 140,099 individuals with a follow-up time of 637,694 patient-years (Figure 1). Our patient population had more women (56%), a mean age of 54.0 (21.2) years, with most living in a major city compared with a regional area (65% versus 35%). The common comorbidities at baseline were hypertension (12%), CKD (7%), and diabetes (7%; Table 1). The mean follow-up time was 4.6 (3.1) years.

**Table 1:** Baseline characteristics of patients with and without acute renal infarct for full cohort and after matching

Variables	Full Cohort			Matched Cohort		
	All Patients (*n*=140,099)	Acute Renal Infarction		All Patients (*n*=714)	Acute Renal Infarction	
		Yes (*n*=119)	No (*n*=139,970)		Yes (*n*=119)	No (*n*=595)
Sex (male)	61,575 (44%)	55 (46%)	61,520 (44%)	349 (49%)	55 (46%)	294 (49%)
**Age, yr**	54.0 (21.3)	65.6 (17.2)	54.0 (21.3)	65.7 (17.6)	65.6 (17.2)	65.5 (17.7)
<50	60,297 (43%)	23 (19%)	60,274 (43%)	134 (19%)	23 (19%)	111 (19%)
50–70	40,631 (29%)	42 (35%)	40,589 (29%)	252 (35%)	42 (35%)	210 (35%)
>70	39,168 (28%)	54 (45%)	39,114 (28%)	328 (46%)	54 (45%)	274 (46%)
**Urbanity**						
Major city	91,595 (65%)	85 (71%)	91,510 (65%)	462 (65%)	85 (71%)	377 (63%)
Regional	48,504 (35%)	34 (29%)	48,470 (35%)	252 (35%)	34 (29%)	218 (37%)
**IRSAD**						
Low	41,174 (29%)	32 (27%)	41,142 (29%)	196 (27%)	32 (27%)	164 (28%)
Middle	44,446 (32%)	45 (38%)	44,401 (32%)	238 (33%)	45 (38%)	193 (32%)
High	54,475 (39%)	42 (35%)	54,433 (39%)	280 (39%)	42 (35%)	238 (40%)
**Comorbidities**						
CKD	10,369 (7%)	26 (22%)	10,343 (7%)	160 (22%)	26 (22%)	134 (23%)
*>60*	129,730 (93%)	93 (78%)	129,637 (93%)	554 (78%)	93 (78%)	461 (77%)
*30–60*	8401 (6%)	22 (19%)	8379 (6%)	132 (18%)	22 (18%)	110 (18%)
*<30*	1968 (1%)	4 (3%)	1964 (1%)	28 (4%)	4 (3%)	24 (4%)
AKI	8191 (6%)	29 (24%)	8162 (6%)	101 (14%)	29 (24%)	73 (12%)
Hypertension	17,027 (12%)	39 (33%)	16,988 (12%)	234 (33%)	39 (33%)	195 (33%)
Diabetes	9975 (7%)	14 (12%)	9961 (7%)	105 (15%)	14(12%)	91 (15%)
Atrial fibrillation	6065 (4%)	24 (20%)	6041 (4%)	140 (20%)	24 (20%)	116 (20%)
CAD	7405 (5%)	18 (15%)	7387 (5%)	86 (12%)	18 (15%)	68 (11%)
CHD	3726 (3%)	15 (13%)	3711 (3%)	63 (9%)	15 (13%)	48 (8%)
Valvular disease	880 (1%)	7 (6%)	873 (1%)	21 (3%)	7 (6%)	14 (2%)
Cerebrovascular	4377 (3%)	11 (9%)	4366 (3%)	45 (6%)	11 (9%)	34 (6%)
PVD	1771 (1%)	20 (34%)	700 (0.5%)	36 (5%)	20 (17%)	16 (3%)
COPD	5092 (4%)	12 (10%)	5080 (4%)	41 (7%)	12 (10%)	41 (7%)
Liver disease	2091 (2%)	6 (5%)	2085 (2%)	18 (3%)	6 (5%)	12 (2%)
Cancer	7731 (6%)	7 (6%)	7724 (6%)	54 (8%)	7 (6%)	47 (8%)
Obesity	974 (1%)	1 (1%)	973 (1%)	8 (1%)	1 (1%)	7 (1%)

Data expressed as number (percentage) or mean (SD). CAD, coronary artery disease; CHD, congestive heart disease; COPD, chronic obstructive pulmonary disease; IRSAD, Index of Relative Socioeconomic Advantage And Disadvantage; PVD, peripheral vascular disease.

### Renal Infarction Incidence

A renal infarction occurred in 119 (0.1%) of the patient population. The crude incidence of renal infarction for our study population was 1.9 per 10,000 patient-years (95% CI, 1.6 to 2.2). There was no difference in the incidence between men (2.0 per 10,000 patient-years; 95% CI, 1.5 to 2.6) compared with women (1.8 per 10,000 patient-years; 95% CI, 1.4 to 2.2) (*P* = 0.5). However, incidence increased significantly by the age group (younger than 50 years: 0.8 per 10,000 patient-years, 95% CI, 0.5 to 1.3; 50–70 years: 2.1, 95% CI, 1.6 to 2.9; and older than 70 years 3.1, 95% CI, 2.4 to 4.0, *P* < 0.001).

### Renal Infarction Risk Factors

There was a higher comorbidity burden in patients who had a renal infarction compared with patients who did not have an episode of renal infarction. Patients with a renal infarction, compared with patients with no renal infarction, were older (mean age 65.6 versus 54.0 years, *P* < 0.001), had higher proportion of background of CKD (22% versus 7%, *P* < 0.001), hypertension (33% versus 12%, *P* < 0.001), diabetes (12% versus 7%, *P* < 0.001), and atrial fibrillation (20% versus 4%, *P* < 0.001; Table 1).

Baseline demographic and comorbidity risk factors for renal infarction are presented in Table 2. After multivariate analysis, the only comorbidities showing a significant risk for renal infarction were older age (by decade odds ratio [OR], 1.17; 95% CI, 1.06 to 1.30; *P* 0.002), history of AKI (OR, 2.41; 95% CI, 1.51 to 3.88; *P* < 0.001), background of atrial fibrillation (OR, 2.55; 95% CI, 1.54 to 4.28; *P* < 0.001), valvular heart disease (OR, 2.88; 95% CI, 1.26 to 6.57; *P* = 0.01), and peripheral vascular disease (PVD; OR, 7.69; 95% CI, 4.54 to 13.4; *P* < 0.001).

**Table 2:** Logistic regression examining variables associated with an increased risk of acute renal infarction

Variables	Univariate Analysis OR (95% CI)	*P* Value	Multivariate Analysis OR (95% CI)	*P* Value
Sex (male)	0.91 (0.64 to 1.31)	0.6	—	—
**Age (decade)**	1.33 (1.20 to 1.46)	<0.001	1.17 (1.06 to 1.30)	0.002
<50	Ref	<0.001	—	—
50–70	2.71 (1.63 to 4.51)			
>70	3.62 (2.22 to 5.89)			
**Urbanity**				
Major city	Ref	0.2	—	—
Regional	0.76 (0.51 to 1.12)			
**IRSAD**				
Low	Ref	0.4	—	—
Middle	1.30 (0.83 to 2.05)			
High	0.99 (0.63 to 1.57)			
**Comorbidities**				
CKD	3.50 (2.27 to 5.42)	<0.001	—	—
*>60*	Ref	<0.001		—
*30–60*	3.66 (2.29 to 5.83)			
*<30*	2.84 (1.04 to 7.73)		—	
AKI	5.20 (3.42 to 7.91)	<0.001	2.41 (1.51 to 3.88)	<0.001
Hypertension	3.53 (2.41 to 5.18)	<0.001	—	—
Diabetes	1.74 (0.99 to 3.04)	0.05	—	—
Atrial fibrillation	5.60 (3.58 to 8.77)	<0.001	2.55 (1.54 to 4.28)	<0.001
CAD	3.20 (1.94 to 5.29)	<0.001	—	—
CHD	5.29 (3.08 to 9.11)	<0.001	—	—
Valvular disease	9.96 (4.63 to 21.4)	<0.001	2.88 (1.26 to 6.57)	0.01
Cerebrovascular	3.16 (1.70 to 5.89)	<0.001		—
PVD	15.9 (9.84 to 25.8)	<0.001	7.69 (4.54 to 13.4)	<0.001
COPD	2.98 (1.64 to 5.41)	<0.001	—	—
Liver disease	3.51 (1.54 to 7.99)	0.003	—	—
Cancer	1.07 (0.50 to 2.98)	0.86	—	—
Obesity	1.21 (0.17 to 8.68)	0.85	—	—

Age used as a continuous variable in multivariate analysis. CAD, coronary artery disease; CHD, congestive heart disease; CI, confidence interval; COPD, chronic obstructive pulmonary disease; IRSAD, Index of Relative Socioeconomic Advantage And Disadvantage; OR, odds ratio; PVD, peripheral vascular disease.

**Table 3:** Risk of outcome after an acute renal infarct episode by full study cohort and matched cohort

Mortality	HR	95% CI	*P* Value	KRT	HR	95% CI	*P* Value
**Full cohort**							
Model 1	2.38	1.77 to 3.19	<0.001	Model 1	17.6	7.86 to 39.5	<0.001
Model 2	1.67	1.25 to 2.24	0.001	Model 2	15.9	7.08 to 35.7	<0.001
Model 3	1.19	0.89 to 1.61	0.2	Model 3	8.41	3.59 to 19.7	<0.001
**Matched cohort**							
Model 1	1.29	0.92 to 1.81	0.1	Model 1	7.99	1.99 to 32.0	0.003
Model 2	1.35	0.96 to 1.91	0.08	Model 2	8.12	1.98 to 33.2	0.004
Model 3	1.11	0.76 to 1.61	0.6	Model 3	10.4	1.96 to 55.2	0.006

Model 1: unadjusted.

Model 2: model 1+age, sex, urbanity, Index of Relative Socioeconomic Advantage And Disadvantage.

Model 3: model 2+CKD, AKI, hypertension, diabetes, atrial fibrillation, coronary artery disease, congestive heart disease, cerebrovascular disease, peripheral vascular disease, chronic obstructive pulmonary disease, liver disease, cancer, and obesity.

Age used as a continuous variable in model 2 and model 3. CI, confidence interval; HR, hazard ratio.

### Mortality Risk

**Figure 2:**
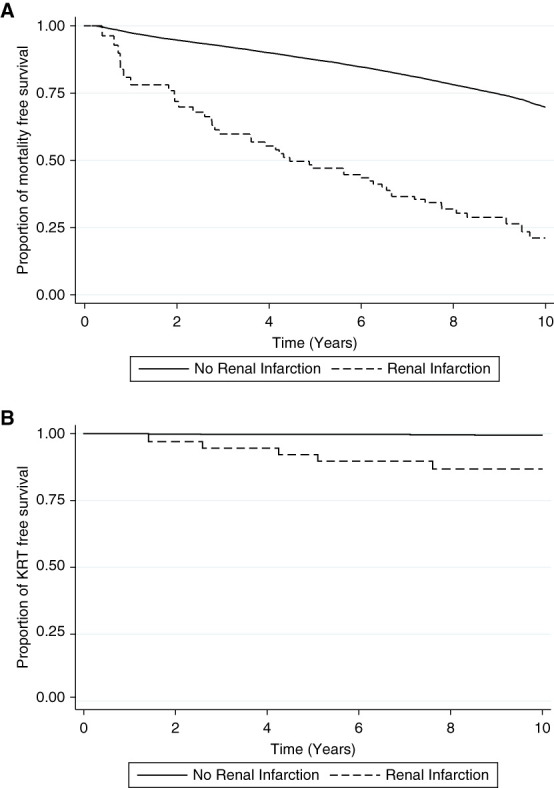
**Kaplan–Meier survival curve.** Kaplan–Meier curve showing time to (A) mortality and (B) KRT according to renal infarction status.

During the follow-up period, 19,001 (14%) patients died. Among patients with a renal infarction, 12 (11%) died within 30 days. The median time to mortality after a renal infarction was 0.8 (interquartile range, 0.08–2.9) years. Mortality was significantly higher in patients with a renal infarction compared with those without (38% versus 14%, *P* < 0.001). In the unadjusted analysis, the risk of mortality was significantly increased after an episode of renal infarction (HR, 2.4; 95% CI, 1.8 to 3.2; *P* < 0.001; Figure 2 and Table 3). However, in the multivariate analysis, after adjusting for age, demographics, and comorbidities, this association was no longer significant (HR, 1.19; 95% CI, 0.89 to 1.61; *P* = 0.2).

### KRT Risk

During the follow-up period, 349 (0.2%) patients commenced KRT. This was more likely in patients with a renal infarction compared with patients with no renal infarction (5% versus 0.2%, *P* < 0.001). In the unadjusted analysis, the risk of KRT was significantly increased after an episode of renal infarction (HR, 17.6; 95% CI, 7.9 to 39.5; *P* < 0.001; Figure 2). Although attenuated, this risk remained significant in the adjusted analysis (HR, 8.4; 95% CI, 3.6 to 19.7; *P* < 0.001).

The six patients with a renal infarct who commenced chronic KRT all started with hemodialysis as the modality. Three patients started chronic KRT within 1 month of the episode of renal infarction, two patients within 6 months, and one patient after 6.5 years.

### Matched Analysis

**Figure 3:**
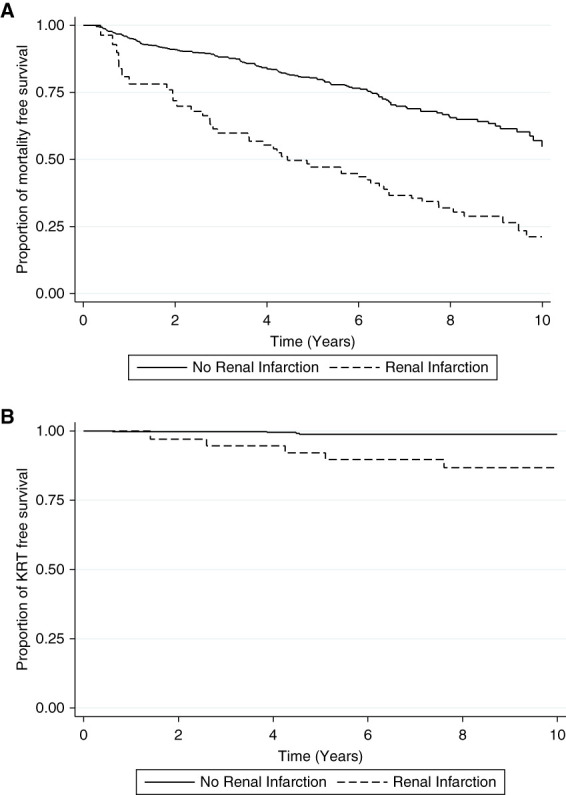
**Kaplan–Meier survival curve.** Kaplan–Meier curve showing time to (A) mortality and (B) KRT according to renal infarction status for the 1:5 matched cohort.

We matched 119 patients with renal infarction to 595 patients with no renal infarction 1:5 on the basis of age (by 5 years), sex, CKD, hypertension, diabetes, coronary artery disease, congestive heart failure, cerebrovascular disease, and atrial fibrillation (Table 1). Similar to the previous analysis, the proportion of mortality was higher after an episode of renal infarction, compared with the matched cohort with no renal infarction (38% versus 24%, *P* = 0.05) and KRT (5% versus 0.5%, *P* < 0.001; Figure 3). There was no significant risk of mortality when comparing patients with renal infarction to the cohort with no renal infarction in both the unadjusted analysis (HR, 1.29; 95% CI, 0.92 to 1.81; *P* = 0.1) and the adjusted analysis (HR, 1.11; 95% CI, 0.76 to 1.61; *P* = 0.6). However, renal infarction remained a significant risk factor for KRT compared with patients with no renal infarction in the unadjusted analysis (HR, 7.99; 95% CI, 1.99 to 32.0; *P* = 0.003) and the adjusted analysis (HR, 10.4; 95% CI, 1.96 to 55.2; *P* = 0.006).

## Discussion

We examined renal infarction in an Australian Local Health District showing an incidence of 1.9 per 10,000 patient-years. This finding confirms that renal infarction is an uncommon and rare disease. We also examined baseline risk factors associated with an episode of renal infarction showing that older age, history of AKI, and a background of atrial fibrillation, valvular heart disease, or PVD significantly increase the risk of experiencing renal infarction. Furthermore, our longitudinal analysis on the outcomes after an episode of renal infarction provides a new perspective on its long-term effects. Notably, after adjusting for comorbidity burden, the data suggest that survivors of renal infarction do not face increased risk of mortality compared with the general population. We did however find a significantly increased risk of commencing chronic KRT. Our results were consistent in adjusted analysis and in a matched cohort analysis. To the best of our knowledge, this is the first study examining this association and comparing outcomes to the general population using a time-to-event analysis. These findings challenge existing perceptions and highlight the potential for positive long-term outcomes under appropriate medical supervision. These insights not only expand our understanding of renal infarction, but also highlight the importance of managing underlying risk factors to mitigate its occurrence and improve patient outcomes.

The prevalence of renal infarction in previous studies varied according to the population examined but consistently confirm that renal infarction is an infrequent event. An older study from 1940 examined 14,000 autopsy patients and found a prevalence of 0.02%.^21^ However, an autopsy might not have identified previous renal infarctions, which may manifest only as renal scars. A more recent cohort study of 18,000 hypertensive patients found a higher prevalence of 0.4%, with an incidence of 1.8 per 10,000 patient-years.^2^ By contrast, lower prevalence rates ranging from 0.004% to 0.015% were found in cohorts of patients presenting to the emergency department.^1,3,5^ These studies are limited by their focus on particular patient groups, which likely contributed to the differences in prevalence estimates.

Studies examining the incidence of renal infarction in the general population are limited. They have shown incidences ranging from 0.3 per 10,000 patient-years for a population-wide Korean cohort^22^ to 0.06 per 10,000 patient-years in two smaller cohort studies.^7,23^ These figures are lower than our estimate of 1.9 per 10,000 patient-years. It is likely that these studies were limited by the shorter follow-up duration and the smaller population in the two cohort studies which likely contributed to the underestimation of the incidence.^7,23^ Our study adds to the limited information available on the prevalence and incidence of renal infarction, showing that the incidence in the general population may be higher than previously anticipated. However, it remains an uncommon event across all studies.

Patients with renal infarction have a high comorbidity burden. Our cohort of patients with renal infarction was older (mean age 66 years) and had a high prevalence of CKD (22%), hypertension (33%), diabetes (12%), atrial fibrillation (20%), and PVD (34%). In particular, atrial fibrillation, valvular heart disease, and PVD significantly increased the risk of a renal infarction event in multivariate analysis. Similar comorbidity burdens were found in other cohorts of patients with renal infarctions, with a mean age of 55–70 years at the time of diagnosis and a high prevalence of hypertension (30%–65%), diabetes (13%–27%), atrial fibrillation (30%–70%), cardiovascular disease (20%–35%), and valvular heart disease (33%).^6,9,13,22,24,25^

The high comorbidity burden becomes relevant when adjusting for the risk of long-term adverse events, such as mortality or commencing KRT. Inpatient mortality after an admission of renal infarction has been reported to occur in 6%–9% of cases,^9,13,26,27^ whereas short-term mortality (within 30 days) can be as high as 9%–13%.^7,28,29^ We found a similar proportion, with an 11% mortality rate within 30 days. However, no previous study has examined the risk of mortality, including long-term mortality, while adjusting for the high comorbidity burden. Our findings confirm that in the unadjusted analysis, an episode or renal infarction increases the risk of mortality, but this association did not persist when adjusted for age and comorbidities.

We found a significantly increased risk of kidney failure requiring KRT in patients with renal infarction compared with the general population or a matched cohort. To the best of our knowledge, this is the first study examining the association between renal infarction and KRT. Previous studies have examined the proportion of AKI after renal infarction and the subsequent development of CKD, both known risk factors for kidney failure. AKI rates reported have been variable, but consistently show that at least one-fifth of patients with renal infarction experience an AKI event. Some studies report lower rates of 20%–39%,^3,5,6,9,11,12,24,28^ whereas others report rates as high as 40%–76%.^7,13,17,29^ The variation is likely a result of the small number of cases in some cohorts and the specific patient population examined.

The rates of AKI requiring acute dialysis are more consistent and an infrequent event, with small cohorts reporting no patients undergoing acute dialysis,^12,26^ whereas larger cohorts report a low prevalence of 4%–6%.^24,29,30^ After an episode of renal infarction, CKD develops in 10%–35% of patients.^3,6,7,9,10,12,24,26,27,30,31^ The rates of kidney failure requiring KRT are lower and more consistent at 2%–8%,^7,9,10,17,24,27,28,32,33^ which is similar to the rate of 5% found our study. To improve the robustness of our findings, we compared the risk of KRT in an adjusted analysis to the normal population and a matched cohort, showing significantly increased risks of HR reaching 8–10. With such large HR, it is important that our results are compared with similar studies to determine validity. The dramatic increase in hazard of KRT after renal infarct may seem initially surprising, but patients who lose renal mass in adulthood in other contexts have demonstrated very high hazard of requiring KRT. For instance, patients undergoing uninephrectomy for live kidney donation have a three- to eleven-fold increase in hazard of KRT,^34,35^ whereas patients undergoing nephrectomy for kidney cancer have a five- to seventeen-fold increased risk of KRT,^36,37^ with the risk increasing three-fold in radical nephrectomy compared with partial nephrectomy,^36^ indicating a dose effect of kidney tissue loss. The markedly increased risk of KRT after renal infarction in our study may be explained by the loss of functioning renal mass similar to above contexts.

A major strength of our study includes the use of a large cohort of patients attending a local health district over a 10-year period, allowing us to capture all hospitalizations and laboratory testings for these patients. Since our local health service covers the entire population of the area, it is likely that we captured all diagnosed episodes of renal infarctions during the study period. The data-linkage also allowed us to incorporate the outcomes of mortality and KRT with a high degree of accuracy. In addition, the large number of patients provided adequate power and allowed for successful 1 to 5 matching with patients with similar characteristics. The large database and comprehensive e-phenotype pf comorbidities facilitated the examination of an infrequent disease, such as renal infarction, to better understand the characteristics, risk factors, and outcomes.

We also acknowledge limitations of our study. For instance, the retrospective nature of our cohort prevents us from proving the direction of causality or the effect of unmeasured confounders. Renal infarction was diagnosed through ICD-10 coding, which may result in some incorrect diagnoses, potentially overestimating our incidence. We were unable to independently review charts to determine accuracy of renal infarction diagnosis or cause, which would have provided important information on prognosis. Reviewing records would also have allowed us to determine the extent of renal infarction, further adding to our findings. We were unable to account for other important variables, such as medication history, which could have strengthened our analysis. Our comorbidity burden for our whole cohort was determined through hospital ICD-10 coding, which could result in misclassification bias, coding biases, or under reporting of certain comorbidities such as obesity or hypertension. Finally, since renal infarction is a rare disease requiring a high degree of suspicion, it is likely that some cases were missed and therefore included in the control group contributing to misidentification bias. We therefore caution that our results should be seen as hypothesis generating, adding to and augmenting the existing literature, with further confirmation needed in other cohorts and datasets.

In summary, we reported the incidence of renal infarction in a large population database and confirmed risk factors for renal infarction. To the best of our knowledge, we are among the first to examine the risk of mortality and KRT after a renal infarction episode, comparing this group with the general population and with a matched cohort. Our findings add novel information, confirming that renal infarction does not increase the risk of mortality when adjusted for the large comorbidity burden. However, we did find a significant risk of KRT, consistent with what other studies on the reduction in renal mass have shown, such as nephrectomy for live kidney donation or for a renal cancer. We would recommend that these findings are confirmed in other settings.

## Supplementary Material

SUPPLEMENTARY MATERIAL

## Data Availability

All data are included in the manuscript and/or supporting information.

## Supplemental Material

Supplemental Table 1. Electronic phenotype comorbidities based on ICD-10 coding.
