# Supplementary material for: Renal Infarction Incidence, Risk Factors, and Risk of Mortality and KRT: A Retrospective Cohort Study
Source: Kidney360. 2025 Feb 18;6(6):947–56. doi: 10.34067/KID.0000000742 (PMC12233841; doi:10.34067/KID.0000000742)
Supplement: SUPPLEMENTARY MATERIAL [file kidney360-6-0947-s001.pdf]

ASN American Society of Nephrology | JASN Journal of the American Society of Nephrology | KIDNEY360 Accessing Our World From Every Angle | CJASN Clinical Journal of the American Society of Nephrology

ASN Journal Disclosure Form

As per ASN journal policy, I have disclosed any financial relationships or commitments I have held in the past 36 months as included below. I have listed my Current Employer below to indicate there is a relationship requiring disclosure. If no relationship exists, my Current Employer is not listed.

S. Aoun Bahous reports the following:
Consultancy: I received payment for consultancy work for Lesaffre.; Research Funding: I am conducting a clinical trial funded by Lesaffre.; and Honoraria: I received honoraria for conferences for Astra Zeneca.

I understand that the information above will be published within the journal article, if accepted, and that failure to comply and/or to accurately and completely report the potential financial conflicts of interest could lead to the following: 1) Prior to publication, article rejection, or 2) Post-publication, sanctions ranging from, but not limited to, issuing a correction, reporting the inaccurate information to the authors' institution, banning authors from submitting work to ASN journals for varying lengths of time, and/or retraction of the published work.

Name: Sola Aoun Bahous
Manuscript ID: K360-2024-001122R1
Manuscript Title: Renal infarction Incidence, Risk Factors and Risk of Mortality and Kidney Replacement Therapy: A Retrospective Cohort Study
Date of Completion: January 28, 2025
Disclosure Updated Date: January 28, 2025

ASN American Society of Nephrology | JASN Journal of the American Society of Nephrology | KIDNEY360 Accessing Our World From Every Angle | CJASN Clinical Journal of the American Society of Nephrology

ASN Journal Disclosure Form

As per ASN journal policy, I have disclosed any financial relationships or commitments I have held in the past 36 months as included below. I have listed my Current Employer below to indicate there is a relationship requiring disclosure. If no relationship exists, my Current Employer is not listed.

H. Cheikh Hassan reports the following:
Employer: Lebanese American University

I understand that the information above will be published within the journal article, if accepted, and that failure to comply and/or to accurately and completely report the potential financial conflicts of interest could lead to the following: 1) Prior to publication, article rejection, or 2) Post-publication, sanctions ranging from, but not limited to, issuing a correction, reporting the inaccurate information to the authors' institution, banning authors from submitting work to ASN journals for varying lengths of time, and/or retraction of the published work.

Name: Hicham I. Cheikh Hassan
Manuscript ID: K360-2024-001122R1
Manuscript Title: Renal infarction Incidence, Risk Factors and Risk of Mortality and Kidney Replacement Therapy: A Retrospective Cohort Study
Date of Completion: January 14, 2025
Disclosure Updated Date: September 11, 2024

ASN American Society of Nephrology JASN Journal of the American Society of Nephrology KIDNEY360 Accessing Our World From Every Angle CJASN Clinical Journal of the American Society of Nephrology

ASN Journal Disclosure Form

As per ASN journal policy, I have disclosed any financial relationships or commitments I have held in the past 36 months as included below. I have listed my Current Employer below to indicate there is a relationship requiring disclosure. If no relationship exists, my Current Employer is not listed.

S. Kallab reports the following:
Employer: Lebanese American University

I understand that the information above will be published within the journal article, if accepted, and that failure to comply and/or to accurately and completely report the potential financial conflicts of interest could lead to the following: 1) Prior to publication, article rejection, or 2) Post-publication, sanctions ranging from, but not limited to, issuing a correction, reporting the inaccurate information to the authors' institution, banning authors from submitting work to ASN journals for varying lengths of time, and/or retraction of the published work.

Name: Siba Kallab
Manuscript ID: K360-2024-001122R1
Manuscript Title: Renal infarction Incidence, Risk Factors and Risk of Mortality and Kidney Replacement Therapy: A Retrospective Cohort Study
Date of Completion: January 29, 2025
Disclosure Updated Date: January 29, 2025

ASN American Society of Nephrology
JASN Journal of the American Society of Nephrology
KIDNEY360 Accessing Our World From Every Angle
CJASN Clinical Journal of the American Society of Nephrology

ASN Journal Disclosure Form

As per ASN journal policy, I have disclosed any financial relationships or commitments I have held in the past 36 months as included below. I have listed my Current Employer below to indicate there is a relationship requiring disclosure. If no relationship exists, my Current Employer is not listed.

H. Kilani has nothing to disclose.

I understand that the information above will be published within the journal article, if accepted, and that failure to comply and/or to accurately and completely report the potential financial conflicts of interest could lead to the following: 1) Prior to publication, article rejection, or 2) Post-publication, sanctions ranging from, but not limited to, issuing a correction, reporting the inaccurate information to the authors' institution, banning authors from submitting work to ASN journals for varying lengths of time, and/or retraction of the published work.

Name: Hala Kilani
Manuscript ID: (K360-2024-001122R1)
Manuscript Title: Renal Infarction Incidence, Risk factors and Risk of Mortality and Kidney Replacement Therapy: A retrospective Cohort Study
Date of Completion: February 3, 2025
Disclosure Updated Date: February 3, 2025

ASN American Society of Nephrology | JASN Journal of the American Society of Nephrology | KIDNEY360 Accessing Our World From Every Angle | CJASN Clinical Journal of the American Society of Nephrology

ASN Journal Disclosure Form

As per ASN journal policy, I have disclosed any financial relationships or commitments I have held in the past 36 months as included below. I have listed my Current Employer below to indicate there is a relationship requiring disclosure. If no relationship exists, my Current Employer is not listed.

K. Lambert reports the following:
Employer: University Of Wollongong; Advisory or Leadership Role: All unpaid: Polycystic Kidney Australia, Scientific Advisory Board; Kidney Health Australia Clinical Advisory Board; and Other Interests or Relationships: Member of the executive of International Society of Renal Nutrition and Metabolism and Australasian Kidney Trials Network Peritoneal Dialysis Working Group.

I understand that the information above will be published within the journal article, if accepted, and that failure to comply and/or to accurately and completely report the potential financial conflicts of interest could lead to the following: 1) Prior to publication, article rejection, or 2) Post-publication, sanctions ranging from, but not limited to, issuing a correction, reporting the inaccurate information to the authors' institution, banning authors from submitting work to ASN journals for varying lengths of time, and/or retraction of the published work.

Name: Kelly Lambert
Manuscript ID: K360-2024-001122R1
Manuscript Title: Renal infarction Incidence, Risk Factors and Risk of Mortality and Kidney Replacement Therapy: A Retrospective Cohort Study
Date of Completion: January 14, 2025
Disclosure Updated Date: January 14, 2025

ASN American Society of Nephrology | JASN Journal of the American Society of Nephrology | KIDNEY360 Accessing Our World From Every Angle | CJASN Clinical Journal of the American Society of Nephrology

ASN Journal Disclosure Form

As per ASN journal policy, I have disclosed any financial relationships or commitments I have held in the past 36 months as included below. I have listed my Current Employer below to indicate there is a relationship requiring disclosure. If no relationship exists, my Current Employer is not listed.

J. Mullan reports the following:
Employer: University of Wollongong

I understand that the information above will be published within the journal article, if accepted, and that failure to comply and/or to accurately and completely report the potential financial conflicts of interest could lead to the following: 1) Prior to publication, article rejection, or 2) Post-publication, sanctions ranging from, but not limited to, issuing a correction, reporting the inaccurate information to the authors' institution, banning authors from submitting work to ASN journals for varying lengths of time, and/or retraction of the published work.

Name: Judy Mullan
Manuscript ID: K360-2024-001122R1
Manuscript Title: Renal infarction Incidence, Risk Factors and Risk of Mortality and Kidney Replacement Therapy: A Retrospective Cohort Study
Date of Completion: January 14, 2025
Disclosure Updated Date: January 14, 2025

ASN
American Society of Nephrology
JASN
Journal of the American Society of Nephrology
KIDNEY360
Accessing Our World From Every Angle
CJASN
Clinical Journal of the American Society of Nephrology

ASN Journal Disclosure Form

As per ASN journal policy, I have disclosed any financial relationships or commitments I have held in the past 36 months as included below. I have listed my Current Employer below to indicate there is a relationship requiring disclosure. If no relationship exists, my Current Employer is not listed.

K. Murali reports the following:
Employer: Wollongong Hospital; Consultancy: GSK; and Ownership Interest: A2M.ASX, AFG.ASX, AGL.ASX, CLW.ASX, CXO.ASX, DLI.ASX, DRE.ASX, DRO.ASX, DSK.ASX, EML.ASX, FGR.ASX, FMG.ASX, FRX.ASX, HLO.ASX, ING.ASX, NVX.ASX, OFX.ASX, PLL.ASX, RHC.ASX, SGR.ASX, SM1.ASX, SPK.ASX, TLS.ASX, TPG.ASX, TWE.ASX, WDS.ASX.

I understand that the information above will be published within the journal article, if accepted, and that failure to comply and/or to accurately and completely report the potential financial conflicts of interest could lead to the following: 1) Prior to publication, article rejection, or 2) Post-publication, sanctions ranging from, but not limited to, issuing a correction, reporting the inaccurate information to the authors' institution, banning authors from submitting work to ASN journals for varying lengths of time, and/or retraction of the published work.

Name: Karumathil Murali
Manuscript ID: K360-2024-001122R1
Manuscript Title: Renal infarction Incidence, Risk Factors and Risk of Mortality and Kidney Replacement Therapy: A Retrospective Cohort Study
Date of Completion: January 15, 2025
Disclosure Updated Date: January 15, 2025
