# Supplementary material for: Renal Infarction Incidence, Risk Factors, and Risk of Mortality and KRT: A Retrospective Cohort Study
Source: Kidney360. 2025 Feb 18;6(6):947–56. doi: 10.34067/KID.0000000742 (PMC12233841; doi:10.34067/KID.0000000742)
Supplement: SUPPLEMENTARY MATERIAL [file kidney360-6-0947-s002.pdf]

| Supplemental Table 1: Electronic phenotype comorbidities based on ICD-10 coding | |
|---|---|
| Comorbidity | ICD-10 code |
| Hypertension | I10x- I13x, I15x |
| Diabetes | E08x- E13x |
| Atrial Fibrillation | I48x |
| Coronary Artery Disease | I20x- I25x, Z95.5, Z95.8, Z95.9, T82.2 |
| Congestive Heart Disease | I09.9, I11.0, I13.0, 13.2, I25.5, I42x, I43x, I50x |
| Valvular Heart Disease | A52.0, I05x- I08x, I09.1, I09.8, I34x- I39x, Q23.0- Q23.3, T82.0, Z95.2- Z95.4 |
| Cerebrovascular Disease | G45x, G46x, I60x- I69x, H34.1, G45x |
| Peripheral Vascular Disease | I70x- I73x, I77.1, I77.9, K55.1, K55.8, K55.9 |
| Chronic obstructive pulmonary disease | J40x- J47x J60x J61x- J68x, J70x |
| Liver Disease | B18x K70x- K76x |
| Cancer | C00x- C26x, C30x- C34x, C37x- C41x, C43x, C45x- C58x, C60x- C76x, C86x, C90x- C97x, D00x- D09x |
| Obesity | E66x |
